# Mother Knows Best: Occurrence and Associations of Resighted Humpback Whales Suggest Maternally Derived Fidelity to a Southern Hemisphere Coastal Feeding Ground

**DOI:** 10.1371/journal.pone.0081238

**Published:** 2013-12-09

**Authors:** Jaco Barendse, Peter B. Best, Inês Carvalho, Cristina Pomilla

**Affiliations:** 1 Mammal Research Institute, University of Pretoria, Pretoria, South Africa; 2 Sustainability Research Unit / South African National Parks, Nelson Mandela Metropolitan University (George Campus), George, South Africa; 3 Ocean Giants Program, Wildlife Conservation Society, Bronx, New York, United States of America; 4 Sackler Institute for Comparative Genomics, American Museum of Natural History, New York, New York, United States of America; 5 Faculdade de Ciências do Mar e Ambiente, Universidade do Algarve, Campus Gambelas, Faro, Portugal; 6 Department of Public Health and Primary Care, University of Cambridge, Cambridge, United Kingdom; Texas A&M University-Corpus Christi, United States of America

## Abstract

Site fidelity is common among migratory cetaceans, including humpback whales (*Megaptera novaeangliae*). In the Northern Hemisphere it has been found that fidelity to humpback whale feeding grounds is transferred maternally but this has never been shown for the species in the Southern Hemisphere. We examined this in a unique feeding area off west South Africa using resighting data of 68 individually identified humpback whales by means of photographic (tail flukes and dorsal fins) and/or molecular methods (microsatellite genotyping) over an 18 year span. We found short-term association patterns and recurrent visits typical of other feeding grounds. Males and females had different seasonality of attendance. Significant female-dominated presence corresponded to timing of an expected influx of females on their southward migration from the breeding ground: firstly non-nursing (possibly pregnant) females in mid-spring, and mothers and calves in mid-to late summer. The potential benefit of this mid-latitude feeding area for females is illustrated by a record of a cow with known age of at least 23 years that produced calves in three consecutive years, each of which survived to at least six months of age: the first record of successful post-partum ovulation for this species in the Southern Hemisphere. We recorded association of a weaned calf with its mother, and a recurring association between a non-lactating female and male over more than two years. Moreover, three animals first identified as calves returned to the same area in subsequent years, sometimes on the same day as their mothers. This, together with numerous Parent-Offspring relations detected genetically among and between resighted and non-resighted whales is strongly suggestive of maternally derived site fidelity at a small spatial scale by a small sub-population of humpback whales.

## Introduction

Annual migration is a well-known life-history trait of many cetacean species [1] and migrations of mysticete whales such as the humpback (*Megaptera novaeangliae*) count among the longest in distance of any vertebrate [2], [3]. Cetacean migrations are characterised by strong site fidelity to the same routes, feeding and breeding areas [4]. Site fidelity can occur at a variety of spatial scales [5] and the possible mechanisms that enable whales to return to the same feeding region are thought to include the use of environmental cues, and matrilineal learning [6]. Such ‘cultural transfer’ of specific migratory routes and other behaviours is especially prevalent in social odontocetes e.g. beluga whales (*Delphinapterus leucas*) [7] and killer whales (*Orcinus orca*) [8], [9] that generally display a much greater degree of kin association and form longer lasting and stronger social structures or networks than baleen whales. Even so, maternally directed cultural transfer has also been proposed as a mechanism for sub-populations of several mysticete species to return to specific feeding grounds, including the North Pacific gray whale (*Eschrichtius robustus*), southern right whales (*Eubalaena australis*) [10], [11] and also the humpback whale [12].

Most available information on the social structure and site fidelity of humpback whales originates from long-term individual identification studies conducted at varied spatial scales in the Northern Hemisphere (NH) [13], e.g. YoNAH – ‘Year of the North Atlantic Humpback’ [14]. In the North Atlantic, humpback whales have been found to display only a very low degree of social organization limited to the formation of small unstable groups [15], [16]. On feeding grounds off Alaska they form larger groups that temporarily associate and cooperate when feeding on Pacific herring (*Clupea pallasi*) [16]–[18], while in the Gulfs of Maine [19] and St. Lawrence [20] some individuals have been involved in longer, relatively stable associations sometimes spanning different feeding seasons. Despite these associations and exceptional cases of ‘team’ work when foraging [21] no conclusive proof of social structure has been found on either their summer feeding, or winter breeding grounds [22] and kinship is not thought to be the determining factor for the specific associations seen when engaging in cooperative foraging [15], [17]. During migration, social behaviour relating to breeding, such as ‘mate guarding’ [23] has been described, but again no genetic evidence for social structure has been found [24].

Humpback whales appear to have a polygynous mating system [15], [25] and in terms of associations between individual males, females, and their direct descendants, there is no known paternal care or association between pairs after conception [26]. On average they reproduce every 2–3 years [27] and new-born calves associate closely with their mothers [28], wean at about six months of age [15] and normally become physically independent by the end of their natal year [29]–[33], although some so-called ‘yearling’ calves are known to accompany their mothers for at least a second year [34]. In the absence of social structure, the experience gained over the period of cow-calf association during nursing and weaning is thought to be critical in determining the choice of migratory route [24], [35], prey [36], and feeding area [37] by the calf after independence. Such maternally directed site fidelity has been confirmed by annual returns of calves to the same NH feeding grounds as their mothers [33], [38].

**Figure 1:**
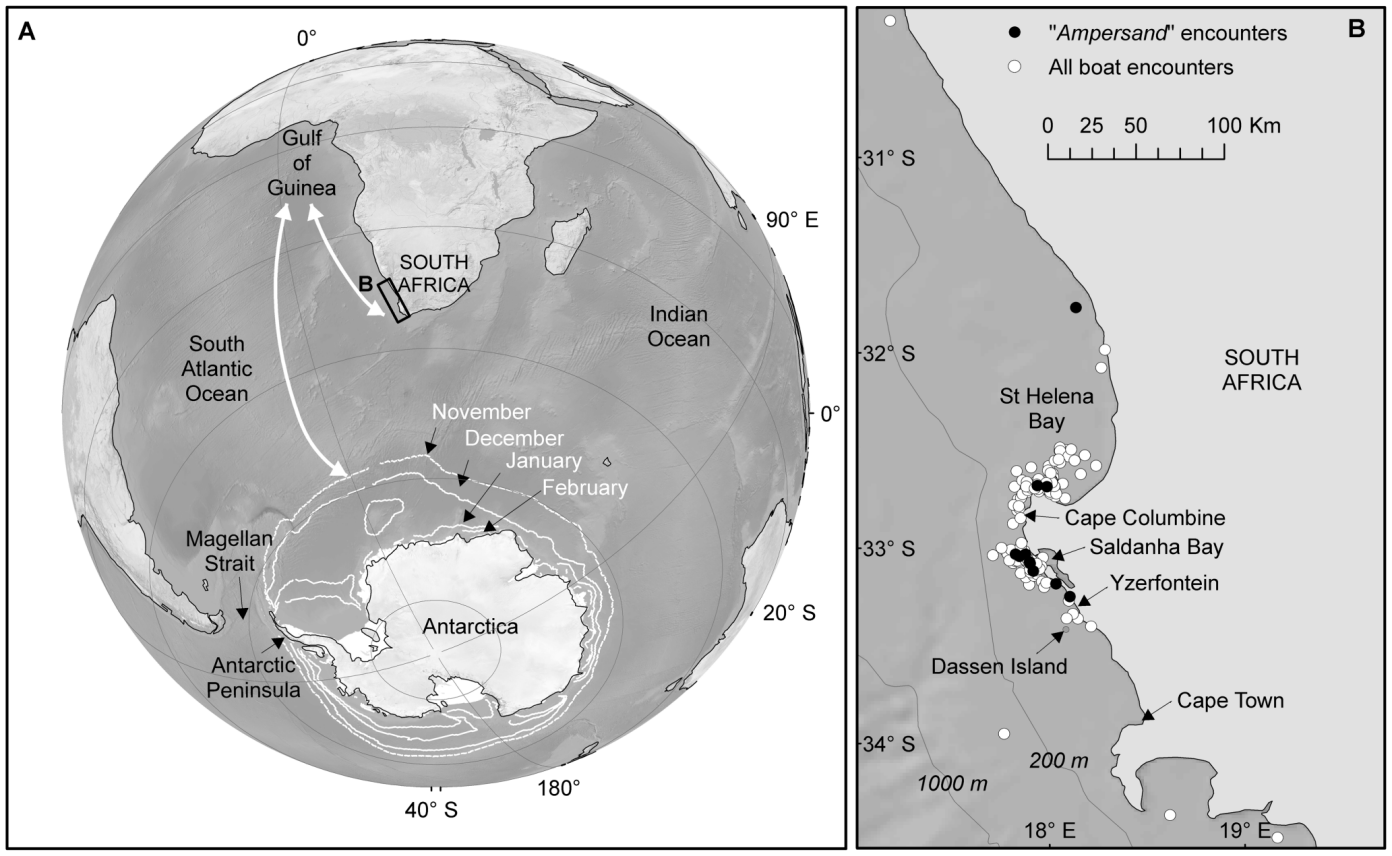
Regions of relevance to West African humpback whales (a) and detail of primary study area and distribution of research effort (b). Key to (a): the Gulf of Guinea where known coastal breeding areas are located; west South Africa migratory corridor/mid-latitude feeding area (rectangle); and Southern Ocean and Antarctica with position of permanent ice shelf (solid white) and ice edges (white lines) in late spring and summer feeding season (Nov – Feb in white font) based on median of measurements, 1979–2000 (data from [100]). White arrows indicate relative difference in possible migration distance from breeding areas to ‘traditional’ Antarctic and west South African feeding grounds. Key to (b): West South Africa and localities of all photo identification and biopsy collections, or mentioned in text (white fill circle  =  boat encounter, black fill circle  =  encounters where #006/*Ampersand* was sighted).

In the context of the Southern Hemisphere (SH), the west coast of South Africa (WSA) is unusual in that it is situated well north of any other comparable feeding area [39]. Furthermore, it serves as both coastal migration corridor [40] and a seasonal near-shore feeding area [41] used during spring and summer by a small component (*ca.* 500 individuals) of the population of humpback whales that breeds in tropical waters off West Africa [42]. The other SH feeding grounds (for the original demarcations see [43]) are typically located at higher latitudes south of the Antarctic Polar Front (at 50°S) and nearer the spring/summer ice edge (Figure 1a), and due to their oceanic nature and remote locations away from human settlements have received only a fraction of the systematic research effort and extensive coverage of equivalent habitats in the NH. Although direct migratory links between low latitude breeding grounds and Antarctic feeding areas have been established in some cases, e.g. [44]–[46], information on the return of individually identified whales to specific feeding localities, or on possible associations or social structure during feeding remains scant, with the possible exceptions of the Magellan Strait [47] and Antarctic Peninsula [48], [49].

Off WSA, an earlier examination of the resightings of individually identified humpback whales has shown multiple annual returns, while sojourns of longer than a month in the same year appeared to indicate temporary residency [42]. These resighted humpback whales thus provide a unique opportunity to investigate whether some aspects of feeding ground utilisation described predominantly from NH locations - such as individual association patterns, maternally derived fidelity and opportunities for cultural transfer - also occur in Southern Hemisphere humpback whales.

## Materials and Methods

### Ethics statement

All work was carried out in terms of permits issued annually under the South African Marine Living Resources Act (Act No. 18 of 1998) administered by the then Department of Environment Affairs and Tourism (South Africa). Specific institutional ethics clearance was not required prior to 2004, and was subsequently approved by the Animal Use and Care Committee, University of Pretoria (Clearance Number: AUCC040405-010a).

### Data collection and encounter database

We compiled a database for individually identified humpback whales from all available photographic and genetic data collected off the west coast of South Africa (see Figure 1b for localities) from February 1983 to January 2008; full details of the collection periods, areas, and effort are described in [42]. In summary: prior to 1993 there was no dedicated whale research effort in the region with only 32 whales identified from 143 pictures and 1 biopsy on 9 different days, all from incidental or opportunistic sightings. From 1993 onwards there were several longer studies dedicated to humpback whales, Heaviside's dolphins (*Cephalorhynchus heavisidii*), or southern right whales (see [40]–[42] and citations therein), which added 257 individuals (based on 215 biopsies and 1,677 pictures) collected on 126 different days. The majority of data were collected in 2001–2007 at Saldanha and St Helena Bays when a boat was available ( =  boat days) either during boat transects or aided by continuous shore-based searches, for a total of 372 days (on average 53 boat days/year) [41], [42]. Collection effort was not evenly distributed between months with an average of 38 boat days per month. The best surveyed months were October and November (96 and 79 boat days respectively), followed by February (49), March (44), September (44), December (43), and January (38). The months from April to July had the lowest number of boat days (13, 7, 14, and 12 respectively) [42].

All data were collected during vessel ‘encounters’, defined here as a discrete data collection event during which an individual whale, sighted alone or as part of a group (see below), was approached closely (±10 m) from the rear and side. Standard procedures acceptable in the field were followed: tail fluke and dorsal fin identification (ID) photos were taken using SLR film or digital cameras with 200–300 mm zoom lenses, and skin samples from individual whales were collected from the flanks and area behind the hump, using the Paxarms rifle-and-dart system (or crossbows from 2005), designed specifically for cetacean biopsy collection [50]. Disturbance to the whales during photo-identification and biopsy operations was kept to the minimum.

Detailed procedures for matching images, microsatellite genotyping of skin biopsies, and sex determination of sampled animals are described in [42], [51]. All encounters with each individual were retrospectively linked using all available ID-features (pictures and genotype); the resulting combined feature database contains 289 individually identified whales seen during 225 different encounters. We included an additional encounter with two known individuals found through tail fluke matches to the Antarctic Humpback Whale Catalogue (AHWC), but sighted off WSA [42].

### Group characteristics and attendance patterns

Throughout this paper, we use the terms ‘resighted’ and ‘non-resighted’ for individual whales only (see later), while groups containing at least one resighted whale will be referred to as ‘familiar’ and those with none as ‘unfamiliar’.

A group was defined as one or more animals in close proximity (<100 m) that displayed similar or visibly coordinated movement or behaviour [52], [53]. Cow-calf pairs were defined as two closely associated whales (sometimes accompanied by other individuals), one of which was visually judged to be 50% or less of the length of the other (also see below).

Within-season attendance patterns of individual whales were examined (after [32]) where ‘Occurrence’ was defined as the number of times (on separate days) the same whale was seen in a pre-determined time period (e.g. a year or breeding season). ‘Occupancy’ referred to the number of days recorded between the first and last sighting dates within this period, counted from the day after the first sighting; i.e. the whale had to be sighted on at least two separate days in the same period. Because of the known summer presence in the region [54] and the low relative abundance observed in autumn to early winter [41], a ‘seasonal offset’ of three months was introduced to capture the presence of ‘over-summering’ animals. Hence, the time unit we used for this analysis was a 12-month period from 1 April of one year to 31 March in the following year; in this context we refer to this unit throughout as a ‘seasonal cycle’, while ‘year’ indicates a calendar year. It follows that we could only calculate occurrence and occupancy for time periods where there was relatively high searching effort with relatively continuous coverage within a seasonal cycle, that is the years 1993–2006, although each of these had months with no coverage [42].

### Sex composition of groups

We compared the sex-ratio of samples from resighted and non-resighted animals to gauge whether one sex was more likely to be resighted than the other. Furthermore, we calculated an ‘operational’ sex ratio (OSR), differing from the definition of Emlen and Oring [55] – ‘the number of fertilizable females to sexually active males’ - in that not all whales were sexed, and that sexual maturity could not be determined (only known calves of the year were excluded). The OSR was calculated for whales available on a daily basis in the study area, and seasonal patterns examined. Although the seasonality of such a sex ratio had been previously reported [41], this did not take the identity of individual whales into account (except on the same day, when duplicate biopsies were excluded), in keeping with other studies that examined similar ratios, e.g. [56]. Because calculation of this ratio depended on the collection of a biopsy, non-biopsied animals of known sex would not have contributed to the reported ratio. Our revised OSR improves on this, by including individuals of known sex every time they were identified (whether biopsied or not) using the full encounter histories available by employing all available ID-features. This should be more fully representative of the OSR, although by giving more weight to ‘resident’ than ‘non-resident’ individuals it would not be equivalent to sex ratios calculated from catches, for instance. We used the same seasonal groupings as in [41], i.e. late autumn to mid-winter (May, June, and July); late winter (August); early spring (September); mid-spring (October); late spring (November); early summer (December); and mid- to late summer (January and February). Resightings in the same month in different years were added together to obtain a single seasonal sample. We use the term ‘season’ in this paper to refer to the four austral seasons in a meteorological and climatic sense, not to be confused with the ‘seasonal cycle’ described above.

### Individual associations

A resighting occurred when an individual was identified during any encounter subsequent to the first, including on the same day. As such the term ‘encounter’ implies identification, bearing in mind that not all individuals were necessarily photographed or biopsied; hence, the resighting of some individuals may not have been recorded. We encountered 68 humpback whales more than once, and these were analysed to identify any short or long term associations and for any evidence of social structure (Table S1). An association was defined as an occasion where two or more known individuals were encountered together in the same group (n = 60, 8 whales were encountered alone and excluded from this analysis). Associations were examined at the level of encounter to allow for detection of the short (same-day) associations that are known to occur at other feeding grounds (e.g. [57]). This means, however, that multiple resightings of the same animal on the same day (we recorded 30 such instances), or repeated associations on a day (10 instances) would affect the overall weight of individual associations between different days. While data from multiple groups on the same day may not necessarily be independent [58], we believe that the small group sizes, few identified individuals, and generally sparse data warranted this approach.

We imported the dataset into SOGPROG 2.4 compiled version (available at http://whitelab.biology.dal.ca/index.html) in ‘group mode’ format [59]. The half-weight association index (HWI) was selected as it is the most commonly used index to describe cetacean associations [20], [60] and displays less bias when (as here) not all associates are identified [61], [62]. The HWI between two individuals (*a* and *b*) was calculated using the following formula: HWI*_a,b_* = *X*/(*X*+0.5(*Y*_a_−*Y*_b_)), where *X* is the number of sightings during which *a* and *b* were seen together, *Y_a_* is the number of sightings when *a* was seen without *b*, and *Y_b_* is the number of sightings when *b* was seen without *a* [60]. Note that the term *Y_ab_* (the number of times *a* and *b* were seen within the same sampling unit, but not together) that is normally added to *X* in the denominator does not apply with ‘encounter’ as a sampling unit, and is therefore omitted. The individual HWI between pairs, and mean and maximum HWI per individual, were calculated. The association matrix (HWI values, rounded to the nearest 0.1) generated by SOCPROG [62] and associated information on gender was exported as a. VNA file into NetDraw v2.097 [63] for display as a social network diagram, and enhanced in CorelDRAW® 12.

### Test for relatedness

We used 10 microsatellite loci and appropriate statistical methods to infer genealogical relationships between pairs of individuals [64], specifically to (a) confirm Parent-Offspring (PO) relations between observed cow-calf pairs and cows with possible yearling-calves, and (b) identify other PO relationships between every possible pair of samples from resighted and non-resighted whales, regardless of being sighted together.

The relatedness of all genotyped whales including 104 non-resighted, 52 resighted, and 10 strandings from the study area (see later), based on the available microsatellite loci was determined using the computer program ML-RELATE [65]. It calculates a maximum likelihood estimate of relatedness (*r*, the coefficient of relatedness defined as the “expected fraction of alleles that are shared identical by descent (IDB)” [64]) and infers the relationship that is most likely for each pair of animals. This is done by examining the IBD coefficients (*k*_0_, *k*_1_, and *k*_2_) that indicate the probability that two whales share 0, 1, or 2 alleles respectively, under a given relationship. These are compared to *k* values expected for the most common higher relationship categories, namely PO and Full Siblings (FS) both for which *r*≥0.5 (see [65] for equations and statistical details). This method accommodates null alleles and is considered to be more accurate than other estimators [66]. Since we were only interested in PO relationships (the null hypothesis), we simulated 10^6^ genotypes to test the alternate hypothesis of FS with the same software for each pair of PO. For pairs where the PO relationship (i.e. sharing the same mtDNA haplotype and at least one allele in each 10-microsatellite locus) was the most supported this would verify maternity in the case of mother-calf pairs seen together. Where a pair was never observed together, or when together not identified as mother-calf, detected PO could indicate either maternity or paternity; the nature of this relationship was inferred from the available sighting history and sex of each whale. We included stranded individuals because we anticipated that they might include a number of young of the year.

## Results

### Group characteristics and attendance patterns

**Figure 2:**
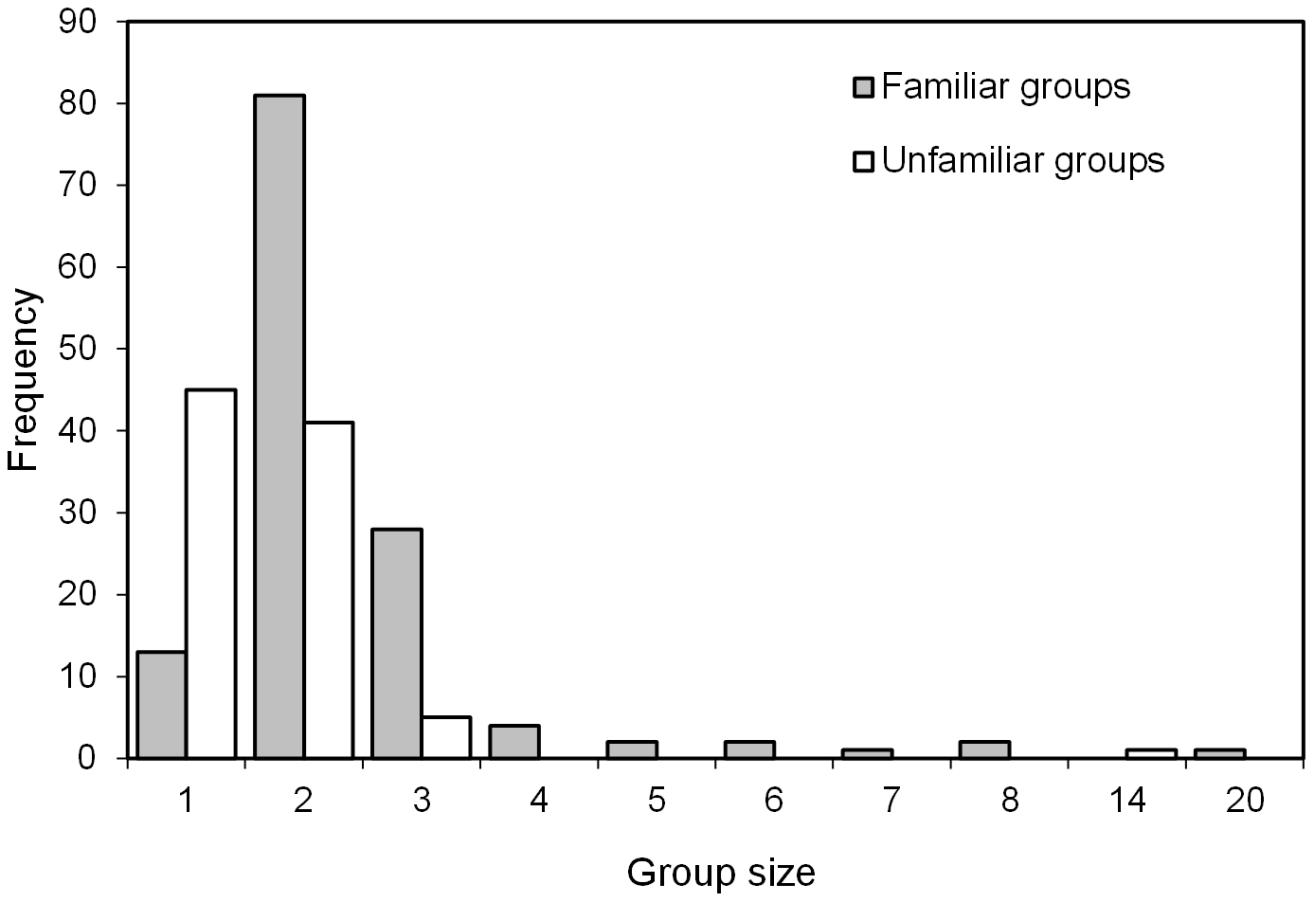
Frequency distribution of group sizes recorded for familiar and unfamiliar humpback whales groups off west South Africa. Familiar groups contained at least one resighted whale, and unfamiliar groups contained no resightings.

The mean overall group size was 2.2±0.12 (SE) (n = 226). The mean size for familiar groups was 2.5±0.17 (SE) (n = 134, range 1–20), significantly larger than the 1.7±0.15 (SE) (n = 92, range 1–14) of unfamiliar groups (*t*-test unpaired: *t* = −3.582, df = 224, *P*<0.001). This difference seemed to arise mainly from the greater proportion of singles (48.9% vs. 9.7%) in the unfamiliar vs. familiar groups: for groups larger than 1, the mean group size of familiar groups (2.7±0.18, n = 121) was not significantly different from the unfamiliar ones (2.4±0.26 n = 47) (*t*-test unpaired: *t* = 1.045, df = 166, *P* = 0.2978). Both familiar and unfamiliar groups had outliers with 20 and 14 individuals respectively (Figure 2); excluding these did not change the non-significant result between these groupings. However, when singles were also excluded, familiar groups remained significantly larger (2.6±0.1, n = 120) than unfamiliar groups (2.1±0.05, n = 46) (*t*-test unpaired: *t* = 2.641, df = 164, *P* = 0.0091). Indeed, all but one of the 13 groups larger than 3 whales contained resighted individuals (Figure 2). Of the 134 familiar groups, 63 were made up entirely of known (resighted) animals, while all others included non-resighted members.

The mean overall occurrence (number of sightings on different days in same seasonal cycle) for the years (1993–2006) was 1.19 times (range: 1–5); single occurrences in a full season were most common (n = 255), followed by twice (27), three (8), four (3), and five times (1). Mean occupancy for whales that were resighted within a full seasonal cycle during all seasons combined (n = 39) was 31.4 days ±9.05 (SE), ranging from 1 to a maximum of 245 days (Table 1). The within-seasonal cycle occupancy when separated by sex (and excluding calves), showed that females (n = 19) had the longest mean occupancy of 36.73 d±13.47 (SE) compared to males (n = 8, 22.75 d±10.41 SE) and unsexed animals (n = 9, 9.56 d±3.96 SE), but not significantly so (Kruskall-Wallis test: *H* = 2.25, df = 2, *P* = 0.3246).

**Table 1:** Occurrence^1^ and Occupancy^2^ of humpback whales off west South Africa resighted on different days during selected season cycles (1993–2007).

		Occurrence			Occupancy (days)	
Seasonal cycle	No. identified	Mean	Max	n	Mean ±SE	Min. – Max.
1993/4	10	2.4	5	5	10±2.92	4–19
1998/9	7	1.29	2	2	7±0	7
1999/2000	10	1.3	2	3	1.33±0.33	1–2
2001/02	78	1.1	2	8	17.86±7.86	1–54
2002/03	90	1.27	4	17	53.24±19.25	1–245
2003/04	22	1.09	3	1	n/a	13
2004/05	43	1.02	2	1	n/a	51
2006/07	34	1.06	2	2	23±15	8–38

1 =  number of different days on which an individual was seen in a seasonal cycle; minimum Occurrence is always one.

2 =  number of days between the first and last sightings in the same seasonal cycle of individuals seen on more than one day.

### Sex composition of groups

We successfully determined the sex for 152 of the genotyped whales (100 non-resighted and 52 resighted individuals). These included 12 different females in cow-calf pairs (10 of these calves were biopsied, yielding 3 females and 7 males), 8 of which were seen more than once, sometimes with the same calf (Table 2). Assuming that the presence of a calf is not independent from that of its mother during its first year, the calves (n = 10) were excluded when analysing sex ratios of samples. The overall sample sex ratio excluding calves (n = 142) was 1 female:0.89 male; for samples from non-resighted individuals (n = 93) it was 1 female:1.07 males and for those from resighted whales (n = 49) 1 female:0.63 male. The sex ratios in non-resighted and resighted individuals were not significantly different (χ^2^, Yates correction  = 1.64, two-tailed, *P* = 0.2) indicating that neither sex was more likely to be resighted. Among the familiar pairs that did not contain calves, we could determine the sex for both members of 20 other pairs, excluding resightings of the same pair on the same day. Most contained a female and male (11), while there were 5 all-female, and 4 all-male pairs. Only 9 non-resighted pairs had both members sexed and comprised 6 mixed, 2 all-female, and 1 all-male. The distribution of mixed-sex, all-female, and all-male groups among familiar and unfamiliar groups did not differ significantly (χ^2^ = 1.34, *P*>0.5).

**Table 2:** Details of humpback whale encounters involving mother-calf pairs off west South Africa of which all the mothers and some calves (in post-natal years) were resighted.

Mother ID	Date	Sighting no.	Group size	Calf ID^1^	Sex of calf	Maternity confirmed^2^	Comments
#6	6 Feb. 1999	10	2	#33	F	yes	Calf of the year
	13 Feb. 1999	1	2	#33	F	yes	Calf of the year
	20 Feb. 2000	2	4	#A	-	-	One of two cow-calf pairs in a group (see #36), both calves of the year
	22 Feb. 2000	4	2	#A	-	-	Calf of the year
	10 Nov. 2001	1	2	#89	M	yes	Calf of the year
	26 May 2002	1	2	#89	M	yes	Calf of the year
#19	04 Mar. 1999	4	2	#38	M	yes	Yearling calf
	14 Feb. 2000	4	2	#39	-	-	Calf of the year
	15 Feb. 2000	7	2	#39	-	-	Calf of the year
#36	20 Feb. 2000	2	4	#B	-	-	One of two pairs in a group (see #6)
#173	10 Jan. 2003	1	2	#C	-	-	Calf of the year
#204	10 Jan. 2003	3	2	#205	M	yes	Calf of the year
	17 Jan. 2003	1	2	#205	M	yes	Calf of the year
	18 Jan. 2003	1	2	#205	M	yes	Calf of the year
#269	21 Nov. 2005	5	3	#E	F	yes	Calf of the year + male escort
	16 Dec. 2006	3	3	#H	-	-	Calf of the year + escort of unknown sex
#286	01 Dec. 2004	3	3	#292	M	no	Calf of the year + male escort
	19 Nov. 2006	1	2	#F	-	no	Calf of the year
#295	11 Jan. 2004	5	2	#D	-	yes	Calf of the year
	23 Nov. 2006	2	3	#G	-	no	Calf of the year + female escort

Identification of calves based on calf size relative to mother, and observation of close association.

1 =  Calves of the year not seen again after natal year are identified by capital letters A-H.

2 =  Maternity confirmed by PO relationship shown in ML-Relate, where samples were available.

**Figure 3:**
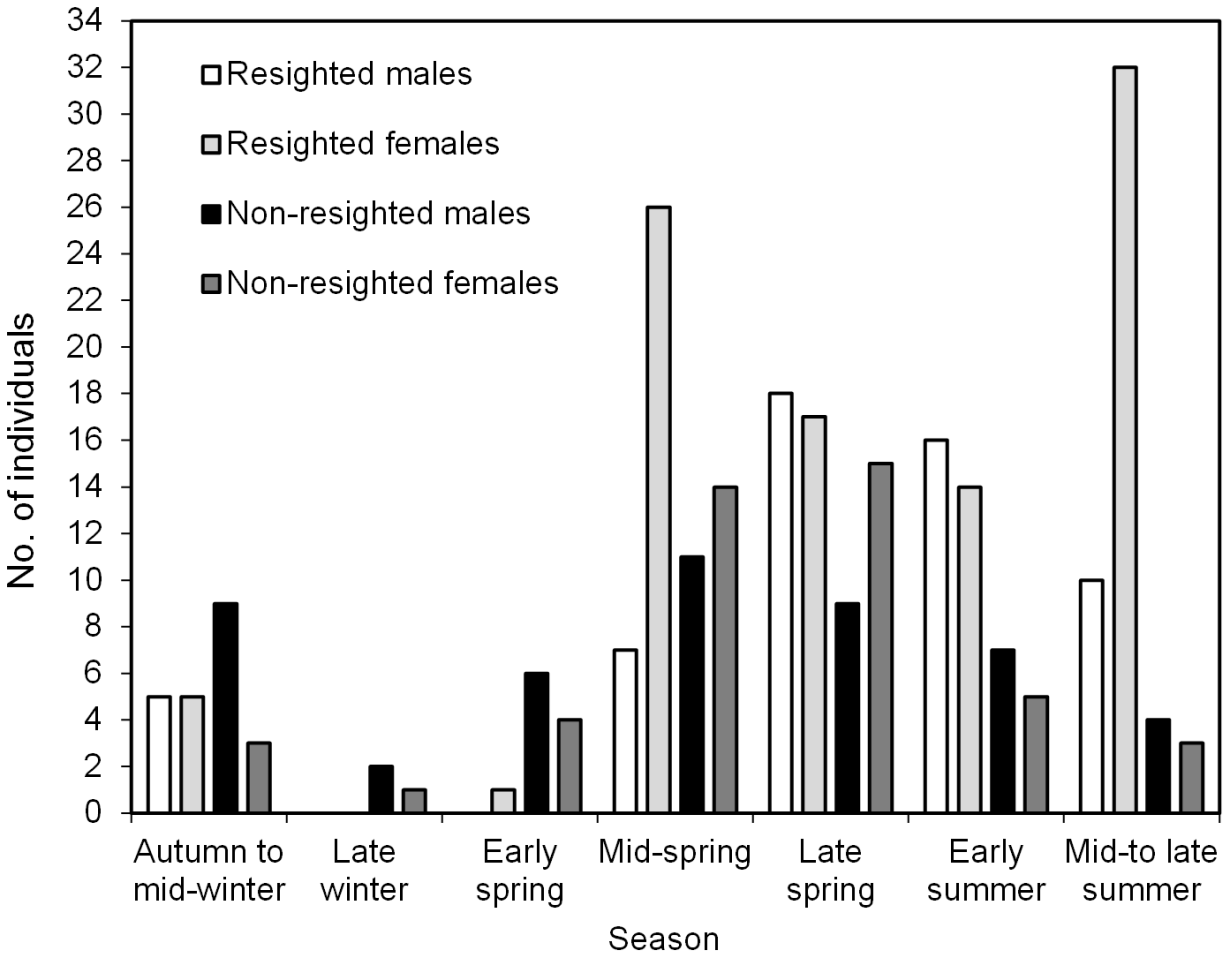
Average daily ‘operational sex ratio’ (OSR) by season in humpback whales identified off west South Africa. Whales were identified from tail fluke and dorsal fin pictures and microsatellites collected during boat encounters. Data shown for both those whales only seen once (n = 93) and those resighted (51 individuals in 151 sightings); calves of the year were excluded.

The seasonal representation of the OSR allows comparison of both the relative contribution of the two sexes, overall, and for resighted and non-resighted whales separately (Figure 3). In all seasonal groupings from autumn through early spring, non-resighted individuals were numerically dominant, and most were male. From mid-spring to mid-late summer the situation was reversed, with resighted individuals numerically dominant in every seasonal grouping, most of which were female. The relative contribution of non-resighted males decreased steadily as the season progressed, after a peak in late winter. This seasonal pattern was similar for non-resighted females. However, the sex-ratio did not deviate significantly from parity in any season except mid-spring (Chi-square: 1F:0.45 M, χ^2^ = 8.35, *P*<0.0039) and mid- to late summer (1F:0.4 M, χ^2^ = 9.0, *P*<0.003), when resighted females predominated among all animals (1F:0.27 M, χ^2^ = 10.94, *P*<0.0009) and only those resighted (1F:0.31 M, χ^2^ = 11.52, *P*<0.0007). We saw very few resighted whales before mid-spring, while their relative contribution (for both sexes) increased from mid-spring onwards. Resighted males from mid-spring to late summer showed virtually the opposite trend to that of resighted females, in that the proportion of males increased between mid- and late spring, and that of females decreased. Males decreased again after early summer, while resighted females were the only groupings to increase sharply after early summer, to nearly two-thirds of all sightings (Figure 3).

### Individual associations and social structure

**Figure 4:**
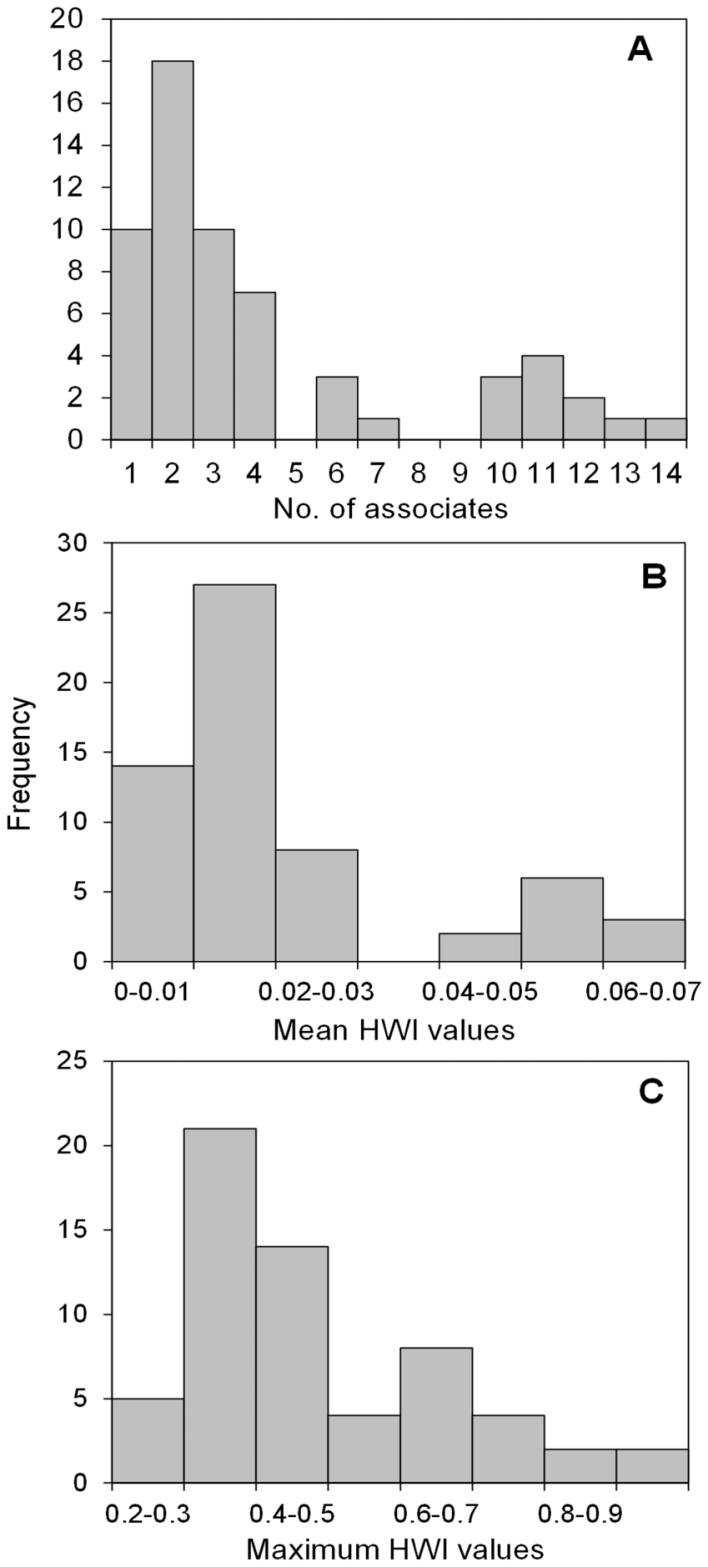
Frequency distributions in individual association parameters for resighted humpback whales off west South Africa. Parameters calculated from full sighting histories in SOCPROG: (a) Number of associates (total number of other resighted whales associated with); (b) Distribution of mean half-weight association indices (HWI); (c) Distribution of maximum HWI between pairs of resighted individual. Values are rounded to the nearest decimal.

Eight individuals were only ever resighted alone or with other non-resighted whales (#50, 75, 176, 207, 240, 269, 282 and 295) and so were excluded from the social structure analysis. The remaining 60 individuals (involving 122 groups on 77 different days) were encountered 2 or more times, together with at least 1 other resighted individual on at least 1 occasion. The frequency distribution of the total number of associates (Figure 4a) with which individual whales were seen (during all encounters), and the raw group resighting data (Table S1) revealed several patterns. The majority of animals were associated with only 2 other known animals, followed by 1 and 3; only 15 individuals associated with more than 4 other known animals (Figure 4a). There was a significant positive relationship between the total number of times a whale was seen and its total number of associates (*r* = 0.359, n = 60, *P* = 0.0049). The number of known animals in a group can also contribute to this, with larger groups containing more potential associates. Furthermore, recurring associations (i.e. with the same individual in more than 1 group, as opposed to ‘new’ individuals) would reduce the total number of associates. For example #6 was seen 11 times and associated with 6 other whales over a period spanning a decade-and-a-half, but never with more than 2 at a time and often the same ones (see later), while #100 was associated with 10 known whales in 3 groups over just 2 days (16/17 December 2001) (Figure 4a; Table S1).

The sighting rates and number of resightings were too low to allow the calculation of a confidence interval (CI) for the measured association indices (a minimum of 15 observations per pair is required to calculate reasonable values, [67] or measures of preferred/avoided companionship). Without CI's, very limited inferences can be made from the magnitude of the mean HWI value, although it should provide a relative indication of the number and recurrence of associations, i.e. a higher mean may indicate that some of the HWI values between pairs were high, or that an individual associated with many others. The mean HWI values ranged from 0.0043–0.0633 with the majority (49 out of 60) below 0.03 and only 11 at or above 0.04 (Figure 4b); these included 7 females, 3 males, and a whale of unknown sex (also see Table 3). Only 3 individuals had a mean HWI>0.06. The lowest maximum HWI value was 0.25 and the highest 1, the latter indicating that some individuals were always seen together. Most maximum HWI values were in the 0.3–0.5 range (35), with only 20 individuals involved in associations stronger than 0.5 (Figure 4c). Mean HWI values of all females (n = 25) and males (n = 21) (including calves) were the same at 0.02.

**Table 3:** Sighting history, number of associations, sex, and maximum half-weight association index (HWI)^1^ of individual humpback off west South Africa.

Individual	Sex	Date first seen	Date last seen	No. of encounters	No. of associates	Max. HWI	Mean HWI
#97	M	16 Dec. 01	21 Nov. 04	4	12	0.57	0.06
#80	F	31 Oct. 01	17 Dec. 01	2	11	0.50	0.06
#100	F	16 Dec. 01	17 Dec. 01	3	10	0.67	0.06
#102	M	16 Dec. 01	17 Dec. 01	2	11	0.50	0.06
#115	F	17 Dec. 01	06 Nov. 02	3	10	0.67	0.06
#118	F	17 Dec. 01	15 Nov. 02	3	12	0.40	0.06
#101	M	16 Dec. 01	29 Nov. 04	8	14	0.55	0.05
#70	F	24 Oct. 01	06 Oct. 03	3	11	0.40	0.05
#29	-	18 Mar. 97	02 Nov. 02	3	11	0.40	0.05
#126†	F	17 Dec. 01	28 Jan. 04	3	10	0.40	0.05
#15	F	05 May 92	20 Jan. 05	8	13	0.50	0.04
#17	F	17 Oct. 93	29 Oct. 05	8	7	0.50	0.03
#18	-	17 Oct. 93	31 Oct. 93	5	6	0.44	0.03
#22	-	27 Oct. 93	31 Oct. 93	3	4	1.00	0.03
#24	-	27 Oct. 93	31 Oct. 93	3	4	1.00	0.03
#107	M	10 Feb. 99	19 Nov. 06	4	4	0.67	0.03

1 =  of whales with mean HWI at or above 0.03.

† =  known mortality.

**Figure 5:**
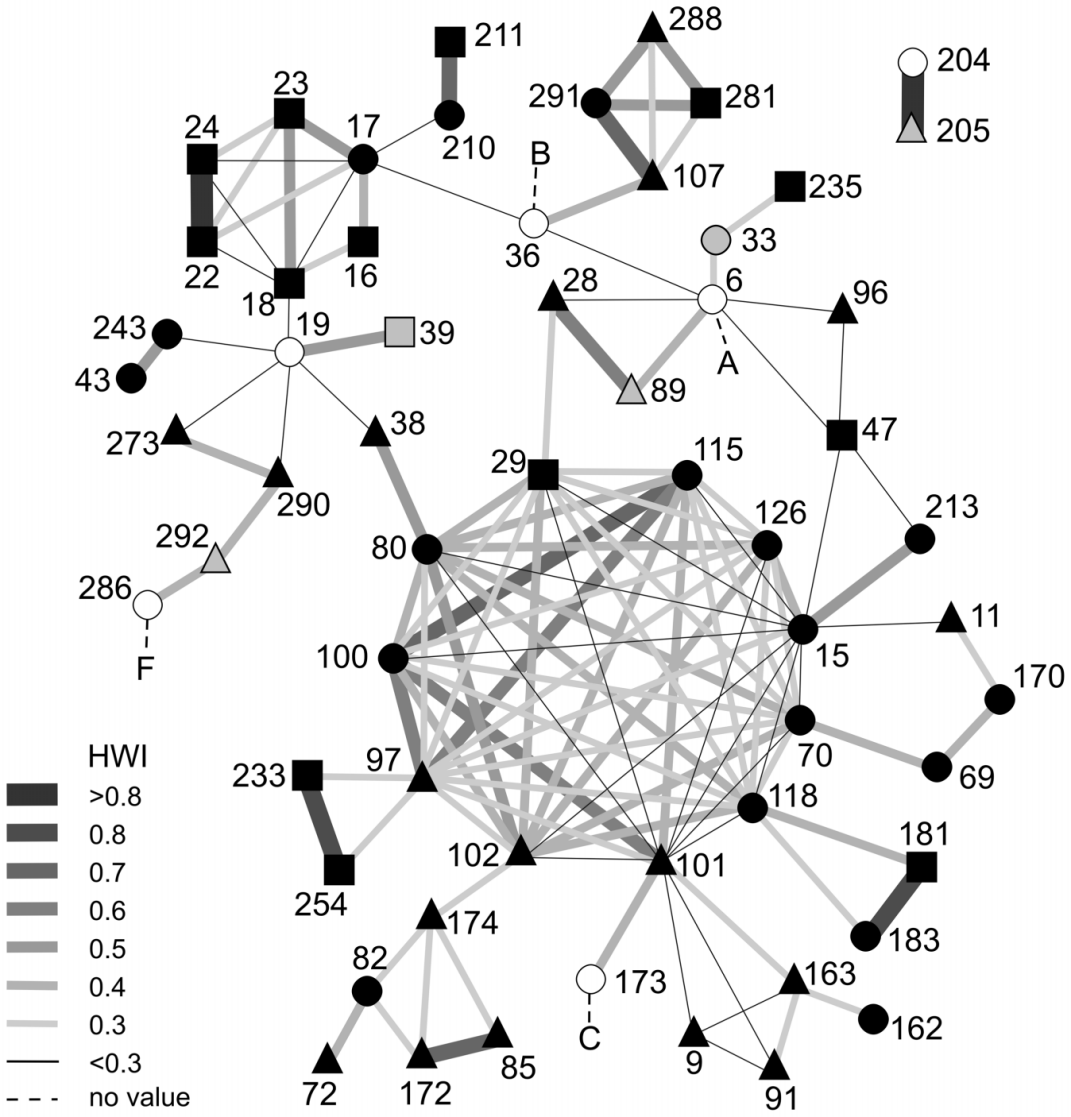
Social network diagram based on half-weight association index (HWI) values calculated between pairs of 60 individually identified humpback whales off west South Africa. Numbers are individual whale identifiers (#); for associations of ≥0.3 the thickness and shading of lines are scaled according to HWI values (rounded to the nearest 0.1); non-resighted calves A, B, C and F (dashed lines) included for information but not used in social structure analysis (also see Table 2). Symbols and colours used: triangles  =  males, black circles  =  females, squares  =  sex not determined, white circles  =  cows, grey fill  =  known calves.

The HWI between a pair highlights the strength of that specific association, bearing in mind that this index is influenced by both the number of times seen together, and sighted separately (see equation above). It is therefore helpful to look at individual cases to put the HWI values into perspective. For example, the HWI between #17 and #36 was 0.13 based on one association on 29 October 2005 (Figure 5; Table S1); they associated with 6 and 2 other individuals, and their mean HWI values were 0.03 and 0.01 respectively. Whale #17 recorded a maximum HWI of 0.5 with #23 (seen together twice in 1993), and a HWI of 0.4 with #16 (also twice in the same year). Whale #36 had the strongest HWI (0.36) with #107; these animals were seen together twice, once on 10 February 1999 and again on 17 December 2001. Compare this to #181 and #183 (Figure 5) both with a mean HWI of 0.02 and maximum of 0.8, but only seen twice (on 30 and 31 October 2002), both times together, once accompanied by another whale (#118). From this it appears that any whale with a mean HWI over 0.3 is worth a closer examination (Table 3), but that it is useful to interpret it together with sighting histories (Table S1).

The social network diagram (Figure 5) prominently features a large ‘cluster’ of 11 whales, 7 females (# 15, 70, 80, 100, 115, 126), 3 males (# 97, 101, 102), and 1 unsexed (# 29), each with 10 or more associates. Virtually all the associations between these individuals occurred during or around the feeding episodes on 16–17 December 2002, either as multiple groups on the same or consecutive days when some were seen in more than 1 group (sometimes together), or where individuals from smaller groups joined to form larger ones (Table S1). Although these associations generally did not last beyond these episodes and HWI values between pairs were predominantly in the range 0.3–0.5, some stronger associations (0.6–0.7) were recorded, e.g. #115 with #100 and #97 (Figure 5). These individuals were among those with the highest mean HWI values (Table 3). Some of the whales (e.g. #15, #29, #80, and #101) present in the larger cluster also associated with whales that did not participate in this grouping but rather in other smaller feeding aggregations, e.g. whales #72, #82, #85, #172, and #174 sighted on 17 October 2002 (Figure 5, bottom left). Other clusters represent discreet instances of multiple associations (smaller feeding groups), such as the Cape Columbine sightings of 17 October–5 November 1993 (Figure 5, top left) and whales #107, #281, #288 and #291 (top centre) that aggregated on 19 November 2005.

Other notable features are more ‘isolated’ pairs sharing much stronger associations (HWI≥0.8). While some of these individuals sometimes did associate with whales from the larger aggregations (for example, the pairs #233 and # 254, or #181 and #183), others never associated with other known whales – such as the cow-calf pair (#204 and #205) that was sighted on several days, always alone (see later). It is noteworthy that the other cows (#6, #36, #173) with calves generally did not associate with multiple whales in the larger aggregations, and seldom had many other associates (excluding their own calves), apart from cow #19 that associated with 5 other whales during 4 different occasions (in 1993, 1999, 2004 and 2005; Table S1). However, these associations tended to be weak (HWI≤0.2) (note also no females with calves present in Table 3).

### Relatedness, cow-calf pairs, and recurring associations

Genetic analysis indicated that 85 individuals (out of 152 biopsied and 10 stranded genotyped whales) were involved in 1 or more Parent-Offspring (PO) relationships: 32 of these were resighted individuals, 50 non-resighted and 3 were stranded animals. Overall we found 65 pairwise PO relationships, 16 between resighted whales, 27 between a resighted and non-resighted whale, 18 between two non resighted whales, one between a stranding and resighting, and three between a stranding and a non-resighted whale. Thirteen of the resighted individuals had 2 or more PO relationships detected: #6 had 4 PO relationships, 6 others had 3 each (including cows #19 and #36) and another 6 had 2 each (including cow #173).

Where genetic samples were available for both, the detected PO relationships confirmed visual determinations that 6 (out of a total of 8 different known whales, resighted on 13 occasions) were nursing mothers with a calf of the year, even though the sample for some calves was collected during a subsequent encounter. We resighted 4 of these on more than one day, sometimes on consecutive days, e.g. #204 and #205 were first seen on 10 January 2003, and again on the 17th and 18th of that month. Incidentally, this calf (a male) was one of the smallest to be observed and associated very closely with its mother during these encounters. The calf had a prominent, somewhat indented, light patch on its left side below the dorsal fin which may have been the result of a recently unfurled dorsal fin, a known neonatal trait [68].

Examples of both short-term associations between a mother and calf (as described above) and much longer periods of association were provided by the individual (#6) with the longest known sighting history. The full sighting histories of this female (nicknamed ‘*Ampersand*’), her offspring and other associates are worth closer examination (Table 2, Figure 5). First identified on 15 January 1988, she was sighted 11 times over a period of 15 years (up to 26 January 2003) in groups that ranged in size from 1–4. During this time we observed her 3 times with 3 different calves, and associated with 4 other resighted animals. The first time she was seen with a calf (#33) was on 6 and 13 February 1999; the calf was identified from left and right dorsal fin, and tail fluke pictures. The following year we sighted *Ampersand* again on 20 and 22 February with a calf (#A) of which the left and right dorsal fins were photographed and did not match those of the previous calf (#33). On the first occasion she was accompanied by another female (#36) also with a calf (#B) (Table 2). Unfortunately neither of these calves was biopsied at the time and there are no subsequent resightings recorded (but see below). On 10 November 2001, we sighted *Ampersand* with a third new calf (#89), that was biopsied and left and right dorsal fins photographed (that did not match those of the previous 2 calves); they were resighted together 6 months later on 26 May 2002. Eight months later, they were seen again, in separate groups associated with other individuals, but also together in a group on 26 January 2003. At this time they were not identified as a cow-calf pair. Both her calves #33 and #89 were resighted in post-natal years, either on the same day (and group) or within a day of their mother, and in close proximity to her sighting (Table 2). Another two PO relationships each were detected for both #6 and female # 36: for the former two resighted females (#69 and #163), and the latter two resighted males (#101 and #174) (Table 4).

**Table 4:** Detected Parent-Offspring relationships among pairs of resighted genotyped humpback whales off west South Africa.

ID 1 {sex}	ID 2 {sex}	LnL(r)	Observation/ Interpretation
**#6** {F}	#33 {F}	−46.62	Cow-calf pair confirmed
	#69 {F}	−49.86	Not seen together, #69 possible weaned calf?
	#89 {M}	−45.64	Cow-calf pair confirmed
	#163 {M}	−48.26	Not seen together, #163 possible weaned calf?
**#19** {F}	#38 {M}	−55.70	Confirm cow and yearling calf
	#91 {M}	−49.45	Not seen together, #91 possible weaned calf?
	**#173** {F}	−55.12	Not seen together, both seen with calves, #19 longer sighting history (1993) so #173 possible calf?
#33 {F}	#172 {M}	−50.50	Not seen together. #33 was calf in 1999, #172 first seen in 2002, offspring or father (of #33 with #6)?
**#36** {F}	#101 {M}	−47.92	Not seen together #36 first seen 1999, #101 first seen in 2001, offspring or father (of #36)?
	#174 {M}	−50.17	Not seen together, #174 first seen in 2001, offspring or father (of #36)?
#291 {F}	#70 {F}	−53.74	Not seen together, no roles assigned, # 70 first seen in 2001 and #291 in 2006. Calf or mother?
	#107 {F}	−52.08	Seen together but no roles assigned, #107 first seen 1999. Calf or mother?
#243 {F}	#162 {F]	−55.53	Not seen together, #162 first seen 2002 and #243 in 2004. Calf or mother?
	#240 {F]	−54.24	Not seen together, #240 first seen 2004. Calf or mother?
**#204** {F}	#205 {M}	−54.98	Cow-calf pair confirmed
**#286** {F}	#292 {M}	−48.31	Cow-calf pair confirmed

Relationships as determined by log likelihood of r (LnL) estimates from ML-Relate (r≥0.5). Individual ID's in **bold** were identified as cows, and those underlined as calves on at least one occasion (also see Table 2 and Figure 5).

We found a similar example of a confirmed calf (#292, a male) being seen 2 years after its natal year (2004), 3 days after its mother (#286) was seen (Table 2) although these sightings were about 30 km apart. This calf was seen in its second year (22 December 2005) with #290 off Cape Town (about 100 km south of Saldanha Bay) (see[42]). In 2006, we saw #286 again with a different calf (Table 2). One more female (#269) was seen with 2 different calves in successive years although neither of the calves was resighted; while females #286 and #295 were seen with calves at 2 and 3 year intervals respectively (Table 2).

The only other example of a recurring ‘stable’ association between years (not by a cow-calf pair) was by the female #36. On 10 February 1999 she was seen with a male (#107) as part of a trio, and they were resighted as a pair nearly 2 years later on 17 December 2001, a day on which one of the feeding episodes occurred. Male #107 was subsequently resighted during another feeding episode (Table 4) and was one of the individuals with a higher mean HWI of 0.04 (Table 3). Female #36 had previously associated with *Ampersand* (on 20 February 2000), when both were accompanied by calves – in the year before she had been seen 3 days before Ampersand, and again seen in her near vicinity on 26 January 2003.

We identified 4 pairs, of which 1 of the animals was noticeably smaller, in the field as cows with possible ‘yearling’ calves. Genetic comparison of these ‘mothers and calves’, and in some instances sex determination (2 of the cows were found to be male!) ruled out maternity in all but 1 case. This pair was seen on 4 March 1999, and later genetically confirmed to be mother (#19) and offspring (#38) (Table 2). The cow was seen with a new calf (#39) the following year, 1 of only 2 of the animals from the 1993 Cape Columbine study [40] to be resighted in later years.

### Record of known mortality

One female (#126) biopsied once before on 17 December 2001 was matched by microsatellite to an animal that was seen floating dead in Yzerfontein harbour on 28 January 2004, and came ashore the next day 1.5 km north of Yzerfontein town (see Figure 1b for locality). The carcass was in poor condition, with most of its skin off. The rostrum was not visible and skull may have been lost; the tongue washed up 100 m down the beach. The cause of mortality could not be established. While still alive, this whale had been resighted on 16 November 2002 and 31 January 2003, matched by dorsal fin pictures. It was also one of the individuals with the highest mean HWI (Table 3).

## Discussion

Despite the small available sample size and limited geographical coverage, our study represents the greatest collection of research effort on any Southern Hemisphere (SH) feeding site and provides unique insights into attendance patterns and individual relations of resighted individual humpback whales, including novel information on the species' reproductive biology in the region.

### Feeding ground only?

The short-term associations, relatively unstable groups, and lack of evidence for long-term social associations or structure seen off west WSA match those on humpback whales in NH feeding grounds [15], [16], [20], [57]. Some of the movement patterns that we observed, such as a mean occupancy of about 30 days per feeding season at a specific site, and individual movement between different sites (e.g. the same whales seen at both Cape Columbine and Saldanha Bay, albeit in different years), are reminiscent of recent findings from the Antarctic Peninsula. Satellite tagged humpback whales feeding there travelled 32 km d^−1^ on average, and employed various foraging strategies, such as short residency times of up to 10 days at specific sites, fluid movements between sites, foraging between adjacent patches (termed ‘commuting’) and movements between more distant areas with different oceanographic conditions (‘ranging’) [48]. The considerable number of non-resighted individuals in spring and summer off WSA suggests that we may be observing both migratory and non-migratory components, a notion supported by the seasonal variation in the directionality and speed of movement as tracked from land, reported in [41]. However, it is difficult to assess how the availability of feeding opportunities may influence the actual migration, i.e. when does it cease to be a migration, as opposed to opportunistic feeding (e.g. [69])?

Migratory behaviour in humpback whales is probably closely linked to the sex, age and reproductive state of the individual whales; factors known to influence the timing of departure or arrival at the termini of a migration path [13], [70]–[72]. Moreover, it has been suggested that the migration functions as a behavioural continuum with breeding grounds, since social behaviours normally associated with breeding such as ‘mate guarding’ [56] and singing [73] by males have been recorded *en route*, or even on feeding grounds [74], [75]. Although singletons were overall the most common grouping off WSA as is the case at NH feeding grounds, the majority of resighted whale groups were in pairs, which is consistent with trends observed during migration elsewhere [24]. In the case of WSA therefore, the apparent semi-residency should probably be interpreted in the context of the area's dual function as migratory route and seasonal feeding area, and the sex and possible reproductive biological needs of the individual whale.

Although numerically we found most of the resighted animals to be female, the sample sex-ratios for non-resighted and resighted whales did not vary significantly from parity. However, the seasonal changes in approximate OSR (i.e. the proportions of males and females available based on full resighting histories of animals) confirmed some of the trends reported previously [41] and could be interpreted as follows: during mid-spring (October), there were significantly more females available, with very few cows with calves. Females without calves and juvenile whales are known to depart first from breeding grounds, and thus should appear first on the southern migratory route, followed by mature males and then cows nursing a calf [71]. The influx of non-lactating females would explain the observed female bias in mid-spring, while juveniles of both sexes presumably also contribute to the numbers. However, the gradual change from a female-dominated sample to one of parity (as opposed to a male-dominated one, see [76]) from October through to December may be explained by the later arrival of (more) males, but also the failure of many of the females (the resighted component) that arrived earlier, to leave the area and continue with their southern migration. This, along with the late arrival of cow-calf pairs, could contribute to the significant peak of resighted females in mid-to late summer.

### Variable habitat utilisation and benefits

The rapid decline in number of males in general and non-resighted males in particular towards the later seasons, suggests that there may be a difference in the way the two sexes utilise this area. The idea of sex-specific habitat use is supported by recent findings in the western South Pacific [77] suggesting that male humpback whales may carry out much more extensive latitudinal and longitudinal movements than females, *inter alia*, by making use of different migratory routes in different seasons, presumably to optimise mating opportunities, which would agree with evidence for variable mating tactics employed by different males [25]. In contrast, females would be expected to favour movements and behaviours that would improve reproductive success and calf survival, i.e. minimise energy expenditure and utilise any available feeding opportunities [77]. Nursing females on breeding grounds typically are less social and associate with fewer other whales compared to non-nursing ones [13], [20], supported here by fewer associates and lower mean association indices recorded for known mothers.

Off WSA, both males and females participated in the feeding aggregations and both sexes were involved in multiple associations. Elsewhere, the predominance of male-female dyads during migration [56] and on feeding grounds has been interpreted as males seeking potential mating opportunities [17], [24]; similarly, instances of male escorts actively following cows with new-born calves (i.e. a female in oestrus) [78]. The sex ratios recorded off WSA for pairs (other than cow-calf pairs) agreed almost exactly with the proportions that would be expected under binomial sampling theory, i.e. 10 pairs with both sexes, 5.5 containing only females, and 4.5 containing only males. This suggests that the pairs represented a random association of sexes: statistically a prevalence of mixed-sex pairs (as found off WSA) would be expected if associations were random with regards to gender.

In the only study to date to examine social structure in humpback whales in detail [20] some evidence was found for long-term associations between mature males and non-lactating females that lasted for up to 6 years. We found one example of such a recurring association between the female (#36) and a male (#107), confirmed not to be its calf, on 10 February 1999, and a re-association nearly 3 years later. Although this may have just been a chance event, this female was seen accompanied by a calf almost exactly a year after the first encounter with #107, indicating that conception must have occurred some 6 months prior to that initial sighting. Incidentally, when resighted with the calf, #36 was associated (in the same group) with the female *Ampersand* (also with a calf); these two were sighted on 2 other occasions in close proximity, once 3 days apart (in February 1999) and once separated by less than 3 hours (on 26 January 2003).

While such sightings do not necessarily prove direct association, they do show strongly corresponding temporal and spatial overlap, with the potential for interaction. Furthermore, the higher incidence of groups larger than 1 containing at least 1 known whale compared to their occurrence as singles suggests a larger degree of sociability by resighted animals. On the other hand, the many Parent-Offspring (PO) relationships detected between resighted and non-resighted whales suggest a much greater degree of relationship than might be expected from the observed number of resightings or the apparent absence of behaviour typically associated with breeding (singing or competitive groups). It is difficult to postulate reproductive segregation when both resighted and non-resighted animals make use of the same migratory route every year, or visit the same feeding ground. Most likely the observations reflect a combination of low whale availability and insufficient sampling effort, both within large groups where not all individuals were identified, or where effort was discontinuous, e.g. due to bad weather [41].

### Opportunities for cultural transfer

It has been proposed that maternally transferred site fidelity to critical habitats (such as feeding areas) in baleen whales may explain the non-recovery of certain sub-populations, or failure to repopulate such habitats, following intensive exploitation [12], [79]. Under this hypothesis, the virtual elimination of a small (sub-) population with a restricted distribution, would, in the absence of immigration from adjoining populations, thwart its recovery indefinitely, since the “cultural memory” to return to the area would have been lost. On this point, the feeding aggregation of WSA provides an interesting case-study, in that it was severely depleted (some 1,300 humpbacks were taken between 1909–1916, see [80]), with limited exchange with adjacent groupings - see discussion in [42]. This probably resulted in their unseasonal occurrence going relatively unnoticed until fairly recently; a phenomenon also observed for humpbacks in the North Atlantic [81] and off Chilé [39], North Atlantic right whales (*Eubalaena glacialis*) [82] and for southern right whales off South Africa [83] and New Zealand [11].

While we can only speculate about the number of humpbacks utilising the WSA feeding ground to have survived whaling, before recovering to an estimated 500 individuals [42] (but see [84] for the potential influence that the presence of transient individuals may have on capture-recapture population estimates at a breeding ground), our findings confirm the presence of mother-calf associations during which such cultural transfer can occur. One of the male-female pairs was confirmed to be a mother with her yearling calf. Such weaned calves have been shown to accompany their mothers [22], but associations beyond the second year have never been documented, although juveniles are known to follow the migratory routes of their mothers. This was confirmed by the 2 calves of *Ampersand* that were resighted and survived beyond their natal years. The first (#33) was seen 3 years after being sighted as a calf, only 1 day before, and within 10 km of where her mother was sighted. The case provided by the third calf (#89) is interesting in that we observed an association lasting at least 6 months, independence by 10 months, and then a re-association at a known age of 14 months, a time by which associations of close proximity should have ceased [26]. Whether this was simply a case of a yearling travelling in the general vicinity of its mother (and briefly re-associating) is unclear. The detection of 10 PO relationships between resighted females and other whales could include their weaned calves not identified, but returning to the area.

Calving intervals of 1 year were established on 3 occasions for 2 different females. Assuming that births peak in August [85], both calves of one female survived to at least 3 months, while all 3 calves of the other female survived to at least 6 months. These are the first recorded annual calvings for southern humpback whales, previously described only from the North Pacific [78], [86] and North Atlantic [37]. The production of 3 calves in 3 successive seasons by a humpback whale has in fact only been reported 3 times before [37], [78]. It has been suggested that females of age ±10 years and older (assuming an average reproductive maturity at about age five, but see [87]) are more likely to achieve a successful annual calving interval than younger animals [78]. This has led to the conclusion that that while postpartum ovulation may be common in humpback whales, only some of the females may be able to successfully maintain the pregnancy, and that this success most likely depends on the availability of adequate prey resources in the season preceding the pregnancy, during the period when the cow is both pregnant and lactating, and during the lactation period for the second calf [78]. All of these criteria were met by *Ampersand*: she was at least 11 years old when first seen with a calf and made use of an additional food source that was available much earlier in the season, and closer to a breeding area, than would be the case for any Antarctic feeding ground (Figure 1a). Incidentally in 2011 *Ampersand* was identified in the field (by JB) from dorsal fin and tail flukes, and appeared to be in very good physical condition (unpublished data). She was seen twice during this research cruise, accompanied by a small calf, on 11 November south of Saldanha Bay and on 19 November at Dassen Island (see Figure 1b), bringing her minimum age to 23 years.

Similar to *Ampersand*, other ‘older’ individuals identified in 1993 and earlier were not only resighted in multiple years, but were conspicuously involved in more associations, were present during summer feeding aggregations (e.g. see #9, #11, #15, #17 and #19 in Figure 5), or had two or more PO relationships with other whales (#6, #9, #15, #19). These included several of the known cows (e.g. #6 and #19), some of their calves (e.g. #33) and also males (e.g. #9). Such long-standing associations or relationships could provide evidence of ‘cultural transfer’, in this case, the use of a specific migratory route and feeding area. Fidelity to feeding areas by southern right whales may be maternally transferred over several generations [10]. The return of at least 3 known calves to the Saldanha Bay area suggests site fidelity by humpback whales at a relatively small spatial scale that was derived from their mother, as suggested in [37].

### Conclusion

We found no persuasive evidence for long-term social associations between humpback whales along the South African west coast; however, the observed short-term associations and movement patterns confirm this to be a feeding area utilised by members of both sexes in mid-spring and summer. The associations seen are more likely a result of groups of animals exploiting a mutual prey resource in a limited area, rather than any co-operative feeding behaviour typically associated with schooling fish prey [88]. In fact, the predominant type of prey taken off WSA, namely euphausiids and other crustacean zooplankton [41], does not occur at the surface [88] nor necessarily require feeding cooperation [16]. Nevertheless, humpback whales are known to be generalist feeders capable of utilising a range of prey types and feeding modes to meet their energy demands [89], and there is historical evidence (stomach contents) of fish-feeding in the region [90].

Apparent non-migratory females contribute to a significant female bias during October and January/February, making use of the feeding opportunities till later in the season than males. Our records of post-partum ovulation and successful pregnancy during lactation are the first for the Southern Hemisphere, and may indicate the possible nutritional advantages that a mid-latitude feeding area offers to females. Although mature males also occur in feeding aggregations, it seems that their attendance in the region may be a combination of more conventional migratory patterns, with some opportunistic feeding included, rather than the exploration of potential mating opportunities. This may explain the departure of most males during mid-to late summer when more lactating females are expected.

Records of post-weaning returns of individuals, and extended associations between mothers and calves, as well as the apparent high incidence of confirmed or potential Parent-Offspring relationships (65 instances from 166 genotyped whales) are strongly suggestive of maternally derived site fidelity. Return to the same feeding area (as its mother) may not necessarily offer detectable reproductive or survival benefits for individuals [92], nor provide evidence for kin-selected associations [93] but it may contribute to reproductive segregation [94], [95] that in turn, could explain the sub-structuring seen in some humpback breeding stocks based on mtDNA analyses, sometimes in the absence of differences in nuclear DNA [96]–[98]. This is of particular relevance to the greater West African humpback population where the breeding stock structure remains unresolved: the genetic differences reported between animals from WSA and Gabon [98], [99] may be partially explained by differential utilisation of the WSA coastal feeding area by the two sexes [51]. Finally, given our small sample size, the potential influence of closely related individuals on results, and the use of few microsatellite loci on assumptions and confidence levels associated with determining higher order relationships [64], [65], [91], a more in-depth examination of the occurrence of PO and FS (from a bigger and more representative regional sample, using more loci) in this sub-population and elsewhere in the region seems warranted.

## Supporting Information

Table S1 **Details of date and time, sighting number, best estimate of group size, and individual identifier (#) of all 134 groups containing resightings of 68 individual humpback whales encountered off west South Africa 1988–2008, including the stranding of a known individual.**
(DOC)
