# Supplementary material for: Mother Knows Best: Occurrence and Associations of Resighted Humpback Whales Suggest Maternally Derived Fidelity to a Southern Hemisphere Coastal Feeding Ground
Source: PLoS One. 2013 Dec 9;8(12):e81238. doi: 10.1371/journal.pone.0081238 (PMC3857176; doi:10.1371/journal.pone.0081238)
Supplement: Table S1 — Details of date and time, sighting number, best estimate of group size, and individual identifier (#) of all 134 groups containing resightings of 68 individual humpback whales encountered off west South Africa 1988–2008, including the stranding of a known individual. (DOC) [file pone.0081238.s001.doc]

# SuppOrting Information

Table S1. Details of date and time, sighting number, best estimate of group size, and individual identifier (#) of all 134 groups containing resightings of 68 individual humpback whales encountered off west South Africa 1988 - 2008, including the stranding of a known individual.

| **Date** | **Time** | **Sight.** | **Grp. size** | **Resighted individuals (#) and sex when known** |
| --- | --- | --- | --- | --- |
| 15 Jan. 88 | - | 1 | 2 | 6_F_ |
| 11 Apr. 89 | - | 1 | 3 | 9_M_ |
| 17 Jan. 90 | 19:05 | 1 | 3 | 11_M_ |
| 05 May 92 | - | 1 | 2 | 11_M_, 15_F_ |
| 17 Oct. –  05 Nov. 93** | - | - | 10 | 16, 17_F_, 18, 19_F_, 22, 23, 24 |
| 18 Mar. 97 | 12:01 | 19 | 2 | 28_M_, 29 |
| 06 Feb. 99 | - | 10 | 2 | **6_F_+33_F_** |
| 10 Feb. 99 | - | 9 | 3 | 36_F_,107_M_ |
| 13 Feb. 99 | - | 1 | 2 | **6_F_+33_F_** |
| 04 Mar. 99 | - | 4 | 2 | 19_F_, 38_M_ |
| 14 Feb. 00 | - | 4 | 2 | **19_F_+39** |
| 15 Feb. 00 | - | 7 | 2 | **19_F_+39** |
| 20 Feb. 00 | - | 2 | 4 | **6_F_+A**, **36_F_+B** |
| 22 Feb. 00 | - | 4 | 2 | **6_F_+A** |
| 27 Mar. 00 | - | 8 | 2 | 43_F_ |
| 29 Mar. 01 | - | 2 | 2 | 9_M_ |
| 05 Apr. 01 | - | 9 | 1 | 47 |
| 24 Oct. 01 | 08:51 | 1 | 2 | 69_F_, 70_F_ |
|  | 11:34 | 2 | 2 | 72_M_ |
| 06 Aug. 01 | 16:57 | 3 | 1 | 50 |
|  | 17:24 | 4 | 3 | 50 |
| 31 Oct. 01* | 15:25 | 4 | 2 | 75_F_ |
|  | 16:59 | 6 | 3 | 38_M_, 80_F_ |
| 03 Nov. 01* | 07:57 | 1 | 3 | 85_M_ |
|  | 11:17 | 2 | 3 | 72_M_, 82_F_ |
| 10 Nov. 01* | 08:00 | 1 | 2 | **6F+89_M_** |
|  | 12:55 | 3 | 2 | 91_M_ |
| 16 Dec. 01* | 08:54 | 2 | 2 | 96_M_ |
|  | 11:10 | 4 | 2 | 15_F_, 97_M_ |
|  | 12:40 | 6 | 2 | 100_F_, 101_M_ |
|  | 14:01 | 7 | 3 | 102_M_, 174_M_ |
| 17 Dec. 01 | 08:12 | 1 | 2 | 36_F_, 107_M_ |
|  | 09:53 | 3 | 4 | 97_M_, 100_F_, 101_M_, 115_F_ |
|  | 11:05 | 4 | 20 | 15_F_, 29, 70_F_, 80_F_, 97_M_, 100_F_, 101_M_, 102_M_, 115_F_, 118_F_, 126_F_ |

| **Date** | **Time** | **Sight.** | **Grp. size** | **Resighted individuals (#) and sex when known** |
| --- | --- | --- | --- | --- |
| 26 May 02 | 13:30 | 1 | 2 | **6_F_+89_M_** |
| 27 Sep. 02 | 16:27 | 4 | 2 | 75_F_ |
| 11 Oct. 02 | 16:34 | 1 | 2 | 33_F_ |
| 12 Oct. 02 | 12:04 | 1 | 1 | 6_F_ |
|  | 17:20 | 3 | 2 | 162_F_, 163_M_ |
| 17 Oct. 02* | 13:06 | 1 | 3 | 170_F_ |
|  | 13:57 | 2 | 2 | 85_M_, 172_M_ |
|  | 14:34 | 3 | 2 | 82_F_, 174_M_ |
|  | 16:28 | 5 | 3 | 176 |
|  | 17:10 | 6 | 2 | 82_F_, 172_M_ |
|  | 17:29 | 7 | 3 | 174_M_ |
|  | 17:29 | 8 | 7 | 85_M_, 172_M_, 174_M_ |
| 30 Oct. 02 | 10:13 | 1 | 6 | 118_F_, 181, 183_F_ |
|  | 12:55 | 2 | 2 | 162_F_ |
| 31 Oct. 02 | 08:31 | 1 | 2 | 181, 183_F_ |
|  | 09:18 | 2 | 2 | 176 |
| 02 Nov. 02 | 13:02 | 4 | 2 | 96_M_ |
|  | 17:07 | 5 | 1 | 29 |
| 06 Nov. 02 | 16:51 | 2 | 2 | 115_F_ |
| 15 Nov. 02 | 11:55 | 2 | 2 | 118_F_ |
| 16 Nov. 02 | 11:16 | 1 | 1 | 126_F_ |
| 13 Dec. 02 | 16:38 | 3 | 2 | 163_M_ |
| 14 Dec. 02 | 10:20 | 1 | 1 | 9_M_ |
|  | 11:02 | 2 | 2 | 101_M_, 163_F_ |
|  | 12:19 | 3 | 4 | 9_M_, 91_M_, 101_F_, 163_F_ |
|  | 09:05 | 1 | 2 | **173_F_+C** |
| 10 Jan. 03* | 12:35 | 3 | 2 | **204_F_+205_M_** |
| 13 Jan. 03 | 09:37 | 1 | 2 | 207_M_ |
|  | 09:51 | 2 | 2 | 210_F_ |
|  | 09:57 | 3 | 2 | 210_F_, 211 |
|  | 12:09 | 4 | 2 | 15_F_, 213_F_ |
|  | 13:21 | 5 | 2 | 210_F_, 211 |
|  | 14:49 | 6 | 2 | 15_F_, 213_F_ |
| 14 Jan. 03 | 12:51 | 1 | 3 | 15_F_, 47, 213_F_ |
|  | 13:02 | 2 | 2 | 6_F_, 96_M_ |
|  | 14:28 | 3 | 3 | 6_F_, 47, 96_M_ |
| 17 Jan. 03 | 09:03 | 1 | 2 | **204_F_+205_M_** |
| 18 Jan. 03 | 09:41 | 1 | 2 | **204_F_+205_M_** |
|  | 11:55 | 3 | 1 | 47 |
|  | 14:20 | 4 | 2 | 207_M_ |
|  | 17:53 | 7 | 2 | 69_F_, 170_F_ |
| 26 Jan. 03* | 09:45 | 2 | 2 | 28_M_, 89_M_ |
|  | 11:22 | 3 | 3 | 6_F_, 28_M_, 89_M_ |
|  | 14:29 | 7 | 2 | 36_F_ |
| 31 Jan. 03 | 18:08 | 8 | 3 | 15_F_, 126_F_ |
| 06 Feb. 03 | 16:17 | 1 | 2 | 11_M_, 170_F_ |
|  | 12:46 | 3 | 2 | 70_F_ |
| 15 Oct. 03 | 12:48 | 2 | 2 | 183_F_ |
| 27 Oct. 03 | 12:32 | 3 | 3 | 233 |
| 25 Nov. 03 | 13:30 | 12 | 1 | 235 |
|  | 15:14 | 14 | 2 | 101_M_ |
| **Date** | **Time** | **Sight.** | **Grp. size** | **Resighted individuals (#) and sex when known** |
| 11 Jan. 04 | 12:29 | 3 | 2 | 240_F_ |
|  | 14:38 | 5 | 2 | **295_F_+D** |
| 15 Jan. 04 | 09:46 | 4 | 2 | 240_F_ |
| 24 Jan. 04 | 11:53 | 1 | 2 | 240_F_ |
| 28 Jan. 04 | Stranded at Yzerfontein | | | 126_F_ |
| 12 Oct. 04* | 11:11 | 4 | 2 | 43_F_, 243_F_ |
| 25 Oct. 04 | 09:03 | 2 | 2 | 204_F_ |
| 08 Nov. 04* | 10:50 | 4 | 2 | 17_F_, 210 |
| 21 Nov. 04 | 14:15 | 1 | 2 | 233, 254 |
|  | 14:20 | 2 | 2 | 33_F_, 235 |
|  | 14:42 | 3 | 8 | 97_M_, 233, 254 |
| 29 Nov. 04* | 10:15 | 6 | 2 | 101_M_, 173_F_ |
|  | 11:57 | 7 | 2 | 101_M_, 173_F_ |
| 01 Dec. 04 | 09:06 | 3 | 3 | **286_F_+292_M_** |
| 02 Dec. 04* | 11:22 | 5 | 2 | 19_F_, 243_F_ |
| 19 Jan. 05 | 09:26 | 1 | 5 | 163_M_ |
| 20 Jan. 05 | 09:02 | 1 | 2 | 15_F_ |
| 23 Mar. 05* | 13:49 | 1 | 2 | 36_F_ |
| 06 Oct. 05 | 12:27 | 4 | 1 | 240_F_ |
| 29 Oct. 05 | 12:01 | 4 | 8 | 17_F_, 36_F_ |
| 21 Nov. 05 | 11:01 | 5 | 3 | **269_F_+E** |
| 24 Nov. 05* | 10:15 | 2 | 3 | 273_M_ |
|  | 11:08 | 3 | 3 | 19_F_, 273_M_, 290_M_ |
| 26 Nov. 05 | 08:45 | 1 | 1 | 207_M_ |
| 22 Dec. 05*** | - | 1 | 2 | 290_M_, 292_M_ |
| 12 Oct. 06* | 09:20 | 1 | 2 | 273_M_ |
|  | 10:58 | 2 | 3 | 281 |
| 01 Nov. 06 | 14:16 | 5 | 1 | 282_F_ |
| 07 Nov. 06 | 12:40 | 22 | 1 | 96_M_ |
| 09 Nov. 06 | 10:55 | 5 | 1 | 282_F_ |
| 19 Nov. 06* | 11:38 | 1 | 2 | **286_F_+F** |
|  | 12:26 | 2 | 1 | 288_M_ |
|  | 13:13 | 3 | 5 | 107_M_, 281, 288_M_, 291_F_ |
|  | 15:25 | 4 | 2 | 107_M_, 291_M_ |
| 22 Nov. 06* | 12:30 | 2 | 2 | 292_M_ |
| 23 Nov. 06 | 08:51 | 2 | 3 | **295_F_+G** |
| 29 Nov. 06* | 10:49 | 5 | 2 | 9_M_ |
| 16 Dec. 06 | 12:57 | 3 | 3 | **269+H** |
| 13 Nov. 07 | 12:21 | 6 | 4 | 9_M_ |
| 05 Feb. 08 | - | 1 | 3 | 213_F_ |

Legend: * = days on which groups were reported to feed or defecate in [41]; ** = summary of 11 encounters at Cape Columbine [40]; *** encounter matched in Antarctic Humpback Whale Catalogue (see [42]); **subscript M** (male **) and F** (female) indicates known sex; **cow-calf** pairs in **bold** **typeface** and joined by **+**, non-resighted calves shown by **capital letters** **A – H** (see Table 2); **shaded cells** indicate individuals or sightings excluded from the social analyses dataset.
